# Recurrent syncopal episodes in a pregnant patient with neurocysticercosis

**DOI:** 10.1016/j.radcr.2023.05.064

**Published:** 2023-06-22

**Authors:** Inderbir Padda, Daniel Aziz, Arun Mahtani, Yashendra Sethi, Sneha Annie Sebastian, Jaime Sexton, Paul Karroum, Daniel Fabian, Matthew Fulton

**Affiliations:** a Department of Internal Medicine, Richmond University Medical Center/Mount Sinai, 355 Bard Ave, Staten Island, NY 10310, USA; b Department of Internal Medicine, Rutgers, Robert Wood Johnson Medical School, New Brunswick, NJ, USA; c Department of Internal Medicine, Government Doon Medical College, Dehradun, India; d Department of Internal Medicine, Azeezia Medical College, Kollam, Kerala, India; e Department of Medicine, St. George's University, University Centre Grenada, West Indies, Grenada

**Keywords:** Cardiology, Case report, Hypotension, Infectious disease, Neurocysticercosis, Seizures, Syncope, Taenia solium, Tapeworm

## Abstract

Neurocysticercosis (NCC) is the most common parasitic infection of the nervous system and acquired epilepsy in low-resource settings due to the pork tapeworm, *Taenia solium*. Humans contract the intestinal infection of the adult tapeworm (taeniasis) through the fecal-oral route after consuming undercooked food, particularly pork or water, contaminated with tapeworm eggs. When the larvae invades the central nervous system (CNS), the infection causes NCC, which often manifests as late-onset seizures, chronic headaches, and intracranial hypertension. We describe a 31-year-old Hispanic multigravida woman from Guatemala, at 33 weeks of gestation, who presented with multiple syncopal and hypotensive episodes prompting a Computed tomography (CT) image of the head revealing multiple small cerebral calcifications indicating NCC. In this article, we highlight the significance of early symptom recognition and diagnostic workup for NCC in areas with diverse immigrant populations. We also discuss the epidemiology, clinical manifestations, and current treatment modalities available for NCC.

## Introduction

NCC is a parasitic infection of the CNS caused by the larval stage *(Cysticercus cellulosae)* of the adult pork tapeworm *Taenia solium* after ingesting undercooked pork. *Taenia solium*, is endemic in most low- and middle-income countries (LMIC) where they present a significant health burden to the community [1,2]. Lately, evidence reports an increase in pork tapeworm infections in developed nations. This is largely because of the high inflow of immigrants from endemic regions. Although accurate incidence data in the United States is not available, NCC incidence ranges from 0.2 to 0.6 cases per 100,000 general population and 1.5-5.8 cases per 100,000 Hispanics [3]. It has become a major public health problem due to an increased incidence of NCC associated epilepsy [2]. In Latin America, Africa, and Asia, NCC is commonly encountered with a broad spectrum of clinical manifestations, like; seizures, headaches, nausea, vomiting, fever, and stiff neck [1,2]. Seizures are the most common clinical presentation of symptomatic NCC occurring in 70%-90% of the cases [4]. Headache is documented in over one-third of patients with focal neurological deficits and signs of increased intracranial pressure being found in only 10% of the cases. Findings suggestive of chronic meningitis, encephalitis, vision impairment, neuropathic pain are rare in patients diagnosed with NCC [4].

Diagnosis can be made using brain imaging such as CT, magnetic resonance imaging (MRI), and serologies, such as enzyme-linked immunoelectrotransfer blot (EITB). Factors determining the clinical manifestations, therapeutic approach, and prognosis of NCC include the location of the parasites (cysticerci) in the brain (extraparenchymal versus intraparenchymal), the number, size, and evolutive stage of the parasites [4]. The main treatment goals of NCC include symptom control, eradication of the parasite, and mitigation of the host's inflammatory process [4]. Treatment options include anthelmintic medications such as albendazole, praziquantel, and niclosamide, with praziquantel and niclosamide being the most commonly prescribed.

## Case report

A 31-year-old multigravida Hispanic female from Guatemala, at 33 weeks of gestation, presented to the labor and delivery unit. She had a syncopal episode at the homeless shelter which was witnessed by other residents. She complained of a headache, but denied nausea, vomiting, neck pain, fever, chills, blurry vision or loss of weight. She migrated from Guatemala to the United States 2 years ago with a known history of epilepsy. Her only medication was levetiracetam 500 mg twice daily. She reported a similar history of recurrent syncopal episodes after the birth of her first child 10 years ago. Upon initial examination, vital signs were normal and the patient was in no apparent distress, awake, alert, and oriented to person, place and time, with no focal deficits on neurological examination. Her cardiac and respiratory system examinations were unremarkable. Abdominal examination revealed a gravid uterus. Peripheral pulses were intact with no visible bilateral lower extremity edema.

**Fig 1:**
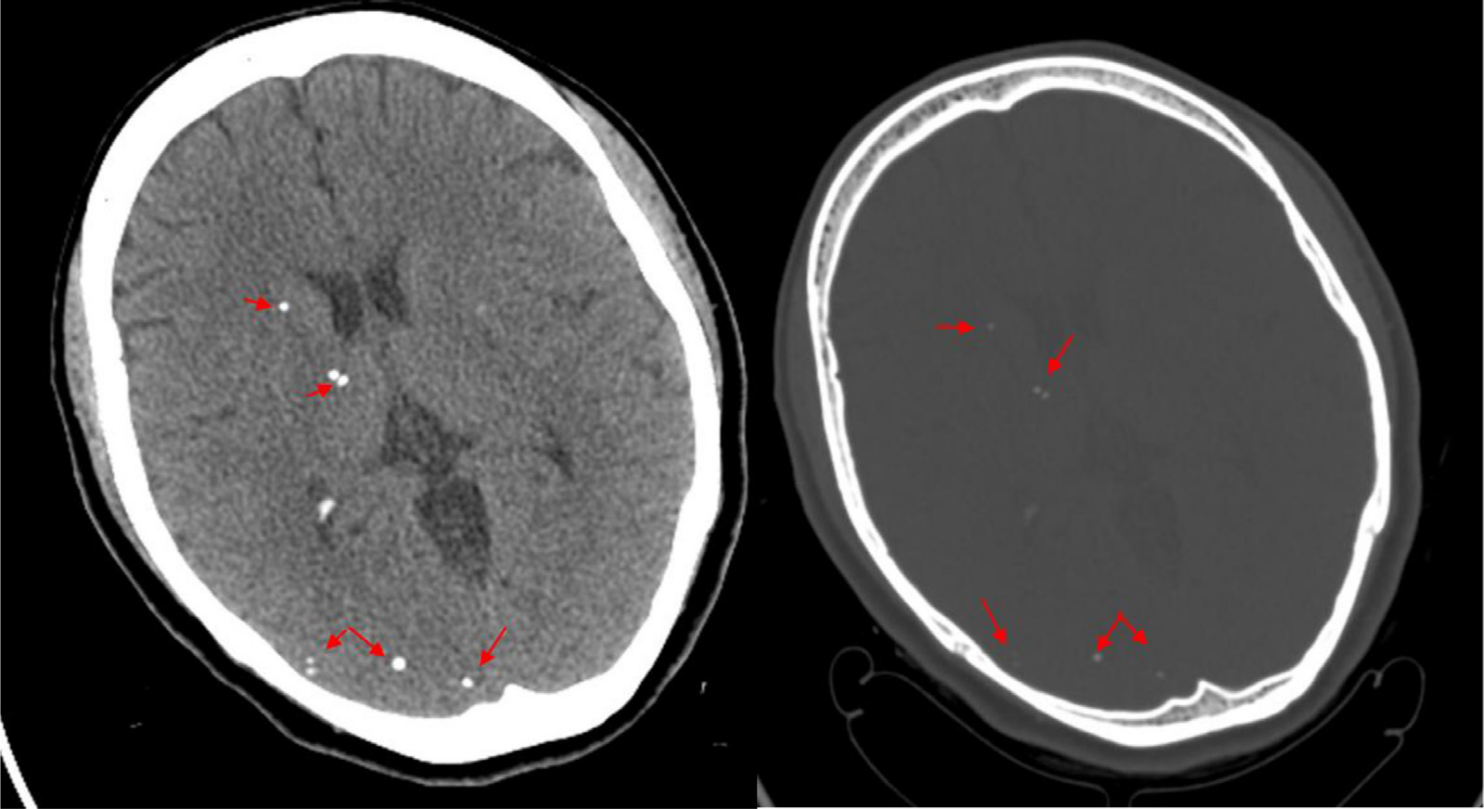
Axial CT of the brain with brain window (left) and bone window (right) showing multiple small cerebral calcifications [Red arrows], without focal mass or lesions. This can be seen in late stage neurocysticercosis or other infections such as toxoplasmosis. Differentials also includes metabolic abnormalities, such as hypoparathyroidism, or possible sequela of prior cerebral infarct.

**Fig 2:**
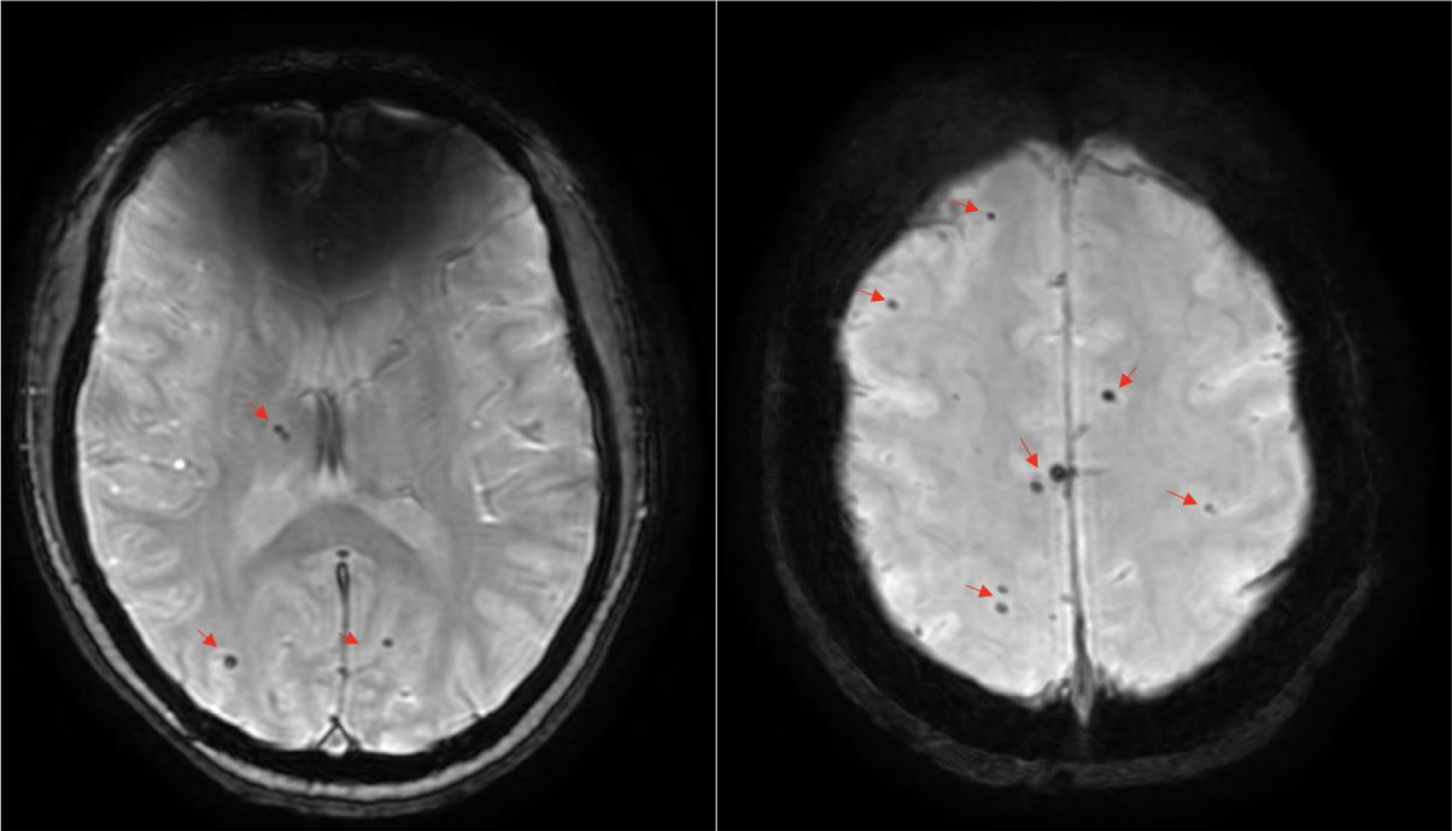
Axial MRI SWAN Sequence (left) and Multiplanar reconstruction (right) showing multiple rounded dark areas in the cerebral hemispheres [Red arrows] corresponding to areas of calcification demonstrated on CT images (Fig 1.). Lesions are noted throughout the cerebral white matter, the thalamus, and the basal ganglia. No other intracranial abnormalities are seen. These findings are consistent with neurocysticercosis.

She was admitted to hospital for further evaluation. During the course of her stay in the hospital she developed recurrent bouts of hypotension which were responsive to fluid bolus. Systolic blood pressure (SBP) was noted to reach as low as 72 mm Hg and a mean arterial pressure (MAP) as low as 52 mm Hg. Upon diagnostic evaluation, a head CT without contrast showed multiple small cerebral calcifications without enhancement or edema indicative of late stage NCC (Fig. 1). An MRI was performed to further assess for active disease, which showed multiple round dark areas in the cerebral hemispheres, corresponding to areas of calcifications demonstrated on CT imaging (Fig. 2). A cysticercosis IgG was ordered and returned negative. She was also tested for SARS-COV-2 RT-PCR (Reverse Transcription Polymerase Chain Reaction), syphilis IgG, Hepatitis-B surface antibody, Hepatitis-C antibody, HIV antigen/antibody, tuberculosis QFT (QuantiFERON-TB) and ab, Respiratory Syncytial Virus RT-PCR, influenza RT-PCR, and rubella antibody, which all returned negative/nonreactive. During the course of her hospital stay, the patients' Ob/Gyn evaluation was unremarkable and syncope secondary to obstetrical complications was ruled out. The decision to treat her latent NCC was deferred as anthelmintics are not indicated in patients with late stage nodular calcifications. Symptomatic treatment for her seizures with levetiracetam 500 mg twice daily was continued and the patient was responsive to fluid boluses for her hypotensive episodes.

## Discussion

NCC is the most common parasitic infection of the nervous system in developing countries including Central America, South America, as well as parts of Africa and Asia [5]. NCC develops from the ingestion of undercooked pork with tapeworm (*Taenia Solium*) cysts known as cysticercus cellulosae*.* The lifecycle of tapeworm involves 2 hosts: the humans and the pigs. Humans are said to be the definitive host of *Taenia Solium (*as the adult worms can reside in the intestinal tract) and they acquire intestinal illness (taeniasis) from pigs, the intermediate hosts, by eating raw pork contaminated with live cysticerci (encysted larvae) or by consuming other contaminated food or water containing T. solium eggs, or through autoinfection. When infected, humans shed the infectious eggs in their feces which, when consumed by either of the hosts, continues the life-cycle, humans shed the infectious eggs in their feces which, when consumed by either of the hosts, continues the lifecycle NCC occurs when these larvae reach the brain and seed as cysts and produce these inflammatory lesions. When cyst formation takes place in the brain due to seeding of the larvae, it is termed as NCC. This is a major cause of adult-onset seizures in developing countries and as of late, in developed countries because of the increased migration from endemic areas [6]. Nearly one-third of seizure disorders are associated with NCC and an estimated 50% of people worldwide have been infected [7]. The prevalence of the disease in Hispanic patients in the US was found to be 9%-13.5% with 60% of patients also being of child-bearing age between 18 and 44 years old [8].

Symptoms of NCC are nonspecific with most patients presenting with seizures, headache, nausea, vomiting, fever, or stiff neck. Diagnosis of NCC during pregnancy is difficult as it presents similar to eclampsia, but clinicians should have a high degree of suspicion based on the clinical history. Imaging should be performed early in pregnancy if NCC is suspected. It is often diagnosed clinically with neuroimaging (CT/MRI) and confirmed with EITB. When it comes to identification of small noncalcified lesions, MRI is more sensitive and should be the modality of choice. CT scan has a higher sensitivity for detection of calcified lesions, but is not as sensitive in detection of subarachnoid and intraventricular lesions. The safest imaging during pregnancy is MRI without gadolinium, as this has not been found to be teratogenic. If CT needs to be performed, it is recommended to use narrow collimation, wide pitch, and shielding [9]. In our case, the head CT demonstrated a nodular calcified stage with no edema or enhancement, indicating a quiescent phase. It is interesting to note here that our case had mostly calcified lesions on imaging, which indicates late/inactive stage of disease contrary to the active NCC which is characterized by viable or degenerating cysticerci.

The test of choice to detect *T. solium* antibodies in the serum is the enzyme-linked immunoelectrotransfer blot (EITB) assay. Sensitivity of EITB varies with the number of lesions present. If only a single lesion is encountered, the sensitivity can decrease to only 50%-60%. ELISA assays are not recommended because of low sensitivity and specificity [10]. Detection of *T. solium* antigen within serum is useful for monitoring response to medication. In our case, the cysticercosis IgG was negative, however that did not deter the diagnosis from being made. Diagnosis was based on family history of tapeworm infection, potential exposure to pork tapeworm, and findings on diagnostic imaging (CT/MRI) (Table 1).

**Table 1:** Summarizes the pathogenesis, clinical manifestations, diagnosis, treatment of NCC [[9], [10], [11]].

Pathogen	Taenia solium
Pathogenesis	Ingestion of undercooked pork or produce containing tapeworm eggs (Taenia solium) which travel to the brain via bloodstream.
Clinical Manifestations	- Seizures - Increased intracranial pressure - Neurological deficits - Headache - Dizziness
Diagnosis	- Head CT Scan - Head MRI - Serological testing: EITB, PCR
Treatment	Viable lesions: Antiparasitics (albendazole, praziquantel) and Corticosteroids(modulate inflammation and reduce edema). Calcified nonviable lesion: Symptomatic treatment (Antiepileptics).

Immunodiagnostic tests can be classified into 2 major types: antigen-based testing and antibody-based testing. Antibodies against cysticercal antigens can be tested on samples such as serum, cerebrospinal fluid (CSF), urine, or saliva. A major disadvantage of this approach is the rate at which false positives may occur. Antibodies do not always indicate an active infection with viable metacestodes. It may result as a false positive because of a resolved infection or exposure to the parasite previously. Another disadvantage is that cross-reactivity can occur with other parasitic diseases, most commonly Echinococcus granulosus [11]. Some other parasitic diseases with cross-reactivity are fasciolasis [12], toxoplasmosis [13], malaria [14], hymenolepiasis [14,15], amoebiasis [14], hepatitis B [14], toxocariasis [11], cerebral tuberculosis [11,16], syphilis [14], and mononucleosis [14,11]. It has been observed that after medication, NCC patients show undetectable levels of IgG4, IgM, and IgA antibodies in their saliva, however antibodies in their serum last longer, regardless of subtype. Immunodiagnosis of cysticercosis can be obtained by utilizing a multitude of mediums such as: low molecular mass (LMM) antigens, excretory/secretory (ES) antigens, total saline extract, antigen B, crude soluble extract (CSE), lentil lectin glycoproteins (LLGPs), vesicular fluid (VF), membrane and scolex extracts, somatic antigens, recombinant proteins, and synthetic peptides [16]. The sensitivity of LLGP-Western Blot is greater than 90%, while the specificity is 100%. This test comprises of SDS-PAGE (Sodium Dodecyl Sulphate-Polyacrylamide Gel Electrophoresis) separation of 7 glycoprotein antigens (50, 42-39, 24, 21, 18, 14, and 13 kDa) and their detection in an immunoblot by CSF serum antibodies [16,17]. However, newer investigations, especially in Indian patients, where almost two-thirds of NCC patients have a single cysticercotic granuloma, have indicated that LLGPs are less sensitive for multiple cysticerci with a sensitivity reported to be between 50% and 80%, and specificity between 94% and 100%. Newer intriguing methods for diagnosing cysticercosis like nanobodies (camelid-derived single-domain antibody fragments) have shown great experimental potential [17,18].

Treatment is dependent on the type of lesions seen on imaging. Viable lesion treatment begins with corticosteroids to help reduce cerebral inflammation. Dexamethasone 0.2-0.4 mg daily prior to, and concurrently with, antiparasitic therapy is recommended as increased intracranial pressure is contraindicated for antiparasitic use. Once intracranial pressures have been normalized, 10 days of combined Albendazole (15 mg/kg/d) plus Praziquantel (50 mg/kg/d) can safely be initiated. While a short course of Albendazole and Praziquantel has been proven to be safe, a longer course of treatment has not been studied [9]. Treatment for calcified nonviable lesions as seen in our case, focuses more on symptomatic relief as the parasites are no longer present within brain parenchyma. Contrary to viable lesions, corticosteroid use should be avoided with calcified nonviable lesions because of the risk of rebound edema following tapering of medication. Patients with acute hydrocephalus need further evaluation for urgent surgical removal of parasites from the ventricle in order to improve CSF drainage. In pregnant and postpartum women with taeniasis, a one-time dose of anti-tapeworm medication niclosamide with mild a laxative is highly recommended to prevent horizontal transmission of cysticercosis to the newborn. The risks and benefits of antiparasitic therapy while pregnant should be discussed with the patient. Most patients prefer to withhold treatment until after delivery because the overall safety of prolonged use of high-dose antiparasitic treatment during pregnancy has limited research available [9]. Antiepileptic medication should be started to prevent further seizures during pregnancy as this can cause hypoxia to the fetus. Caution should be taken with antiepileptic medications especially those on valproic acid due to the incidence of malformations associated during the first trimester of pregnancy. Our patient was treated symptomatically with fluid boluses for syncope secondary to decreased BP and antiepileptics for seizures. Antiparasitics were not administered as our patient was in late stage NCC and therapy with antiparasitics are not indicated.

## Conclusion

This case report describes an unusual clinical presentation of recurrent syncopal episodes secondary to hypotension in a 31-year-old migrant Hispanic female with multiple small calcified cerebral lesions. Alongside standard causes of syncope such as cardiogenic, neurocardiogenic, and orthostatic hypotension, neurologically mediated syncope secondary to an infectious etiology should be considered and evaluated in individuals migrating from endemic regions. As migration is prevalent around the world, we recommend considering neurocysticercosis in the differential diagnosis if it fits the demographic even in developed countries.

## Patient consent

The case information in this manuscript has been provided with informed consent from the patient presented.
